# A Case Report of Hepatocellular Carcinoma in a Non-cirrhotic Patient With Liver Iron Overload Associated With Myelodysplastic Syndrome

**DOI:** 10.4021/wjon611e

**Published:** 2014-01-16

**Authors:** Naruhiko Ikoma, Hiroharu Shinozaki, Akihito Kozuki, Sho Ibuki, Kokichi Sugano, Makio Mukai, Yoshihiro Masuda, Kenji Kobayashi, Yoshiro Ogata

**Affiliations:** a Department of Surgery, Saiseikai Utsunomiya Hospital, 911-1 Takebayashimachi, Utsunomiya, Tochigi 321-0974, Japan; b Department of Surgery, University of Texas Health Science Center at Houston, Houston, Texas, USA; c Tochigi Cancer Center Research Institute, Tochigi, Japan; d Division of Diagnostic Pathology, Keio University Hospital, Tokyo, Japan; e Department of Hematology, Saiseikai Utsunomiya Hospital, Tochigi, Japan

**Keywords:** Hemochromatosis, Iron overload, Hepatocellular carcinoma, Non-cirrhotic liver, Myelodysplastic syndrome, Liver resection

## Abstract

Hereditary hemochromatosis (HH) is associated with an increased risk for hepatocellular carcinoma (HCC) occurring predominantly in patients with cirrhosis at the time of diagnosis. However, HCC in non-cirrhotic liver of patients with secondary hemochromatosis is rare. A 67-year-old man with a history of myelodysplastic syndrome (MDS) was found to have a liver tumor on abdominal computed tomography (CT). On the basis of findings of high levels of serum ferritin and transferrin saturation, and low intensity of liver parenchyma on magnetic resonance imaging (MRI), we made a diagnosis of hemochromatosis. Liver resection was performed and the specimen showed moderately differentiated hepatocellular carcinoma surrounded by non-cirrhotic liver parenchyma with intracellular iron deposition. Multicentric recurrence of liver tumor occurred 2 years after the surgery despite of deferoxamine therapy with well controlled ferritin level. We reported a case of hepatocellular carcinoma in a non-cirrhotic patient with liver iron overload secondary to MDS. It demonstrated the importance of early detection and initiation of treatment of iron overload in preventing HCC in MDS patients, even among Asian population.

## Introduction

While Caucasians have a high prevalence rate of hereditary hemochromatosis (HH), the Japanese have a low prevalence rate of the germline HFE mutation because of the rarity of HH in Japan [1]. On the other hand, secondary iron overload syndrome is occasionally seen in patients with liver diseases, hematological disorders including myelodysplastic syndrome (MDS), and polytransfusion. Although HH patients have been reported to have an increased risk of hepatocellular carcinoma (HCC) at the time of cirrhosis diagnosis, HH patients without cirrhosis have been rarely found to have HCC [2, 3]. Moreover, HCC in patients with secondary iron overload syndrome has been rarely reported. We report a case of HCC and its multicentric recurrence in a non-cirrhotic MDS patient with secondary liver iron overload. Regarding the fact that less than 2% of HCC develops in a non-cirrhotic liver and almost never in a normal liver [4], this case provides supporting evidence of iron-induced carcinogenesis and shows the importance of early detection of iron overload and initiation of iron removal therapy in MDS patients.

## Case Report

A 67-year-old diabetic Japanese male with a 4-year history of MDS without a history of blood transfusion or ferrotherapy was found to have a liver tumor, which was incidentally detected by computed tomography (CT) when he had pneumonia. He had no contributory family history. The laboratory results indicated macrocytic anemia and normal liver function. Tumor markers including alpha-fetoprotein and PIVKA-II were within the normal limits. The viral markers for hepatitis C and B were negative. The serum ferritin level was high at 2,540 ng/mL, the TIBC and UIBC were 212 and 20 µg/dL, respectively, and the transferrin saturation was 94%.

**Figure 1:**
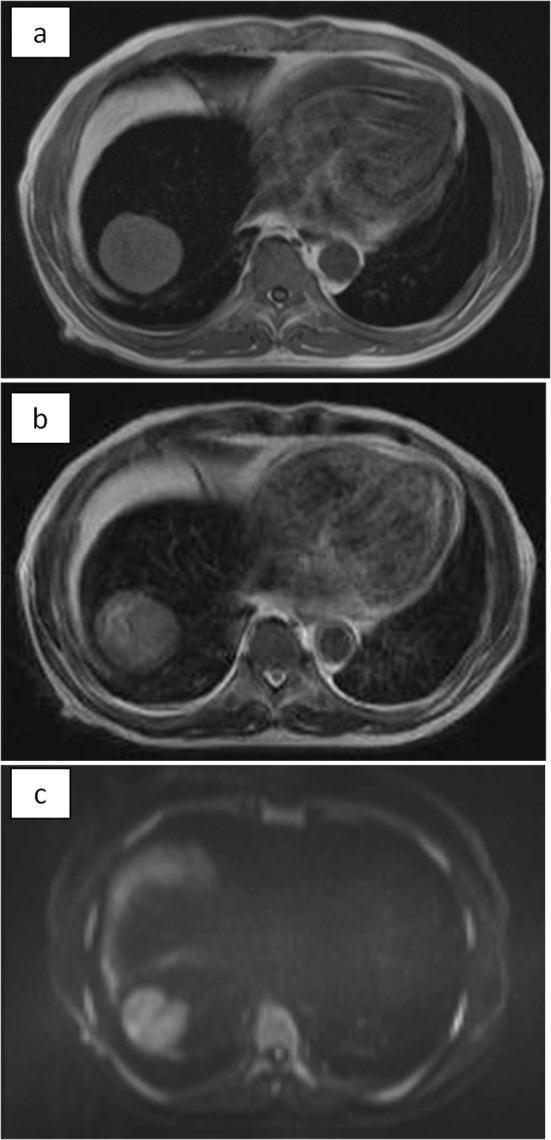
MRI. The tumor showed high intensity on T1 (a), T2 (b), and diffusion-weighted (c) images. The non-tumorous area of the liver showed prominently low intensities both on T1 and T2, indicating extensive iron deposition in the liver parenchyma. On diffusion-weighted image, the parenchyma also showed low intensity due to T2-blackout effect.

The abdominal ultrasonogram showed a low-echoic mass in the right lobe of the liver, and slightly hyperechoic liver parenchyma. The abdominal CT showed a 5.5-cm solitary low-density tumor in segment 8 of the liver, with contrast enhancement in the arterial phase and washout in the delayed phase. The liver parenchyma showed a density lower than that of the spleen. No radiological evidence of liver cirrhosis was identified. On magnetic resonance imaging, the tumor showed high intensities on the T1-, T2-, and diffusion-weighted image. The liver parenchyma showed prominently low intensities on the T1- and T2-weighted images, indicating extensive iron deposition (Fig. 1).

**Figure 2:**
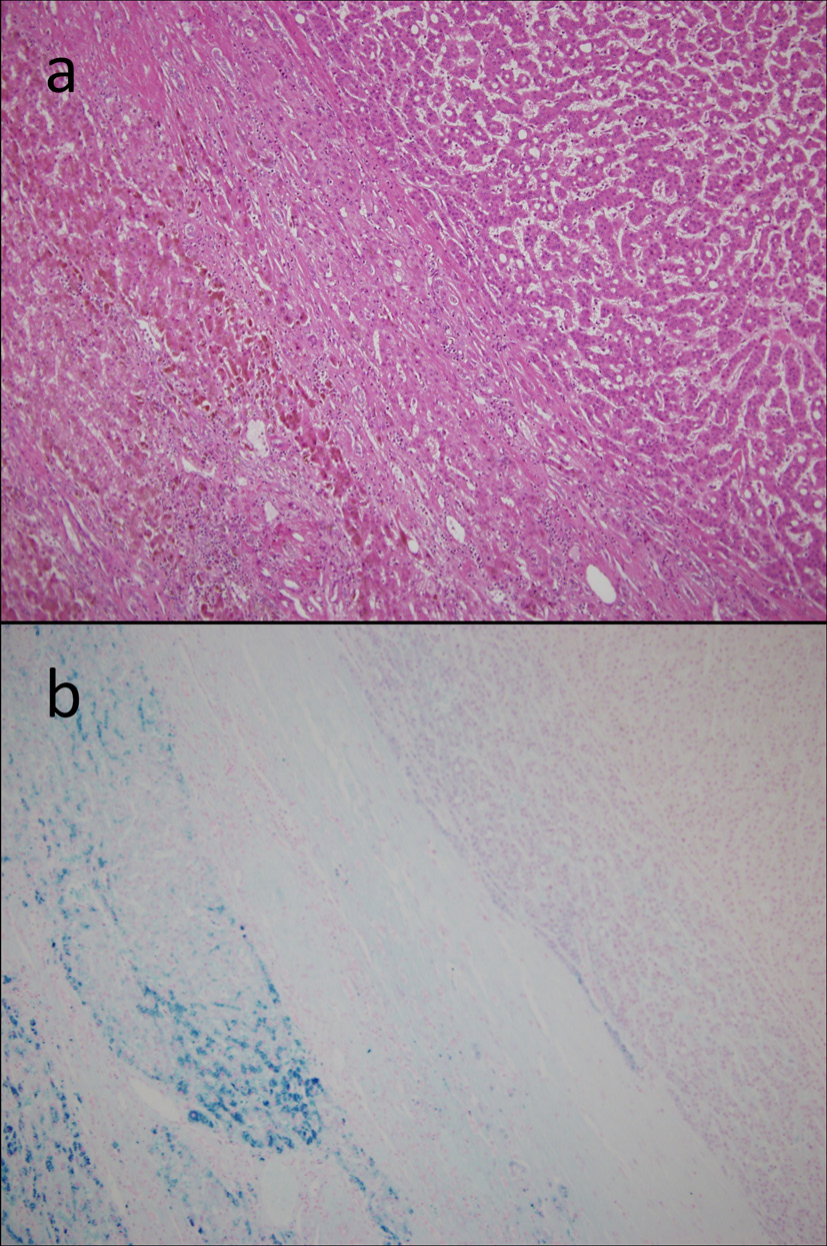
Resected specimen. On microscopic examination, the tumor had the features of a moderately differentiated hepatocellular carcinoma with trabecular and solid patterns, surrounded by non-fibrotic liver parenchyma on the H&E staining (a). The iron stain showed an extensive intracellular iron deposition in the liver parenchyma but none in the tumor itself (b).

**Figure 3:**
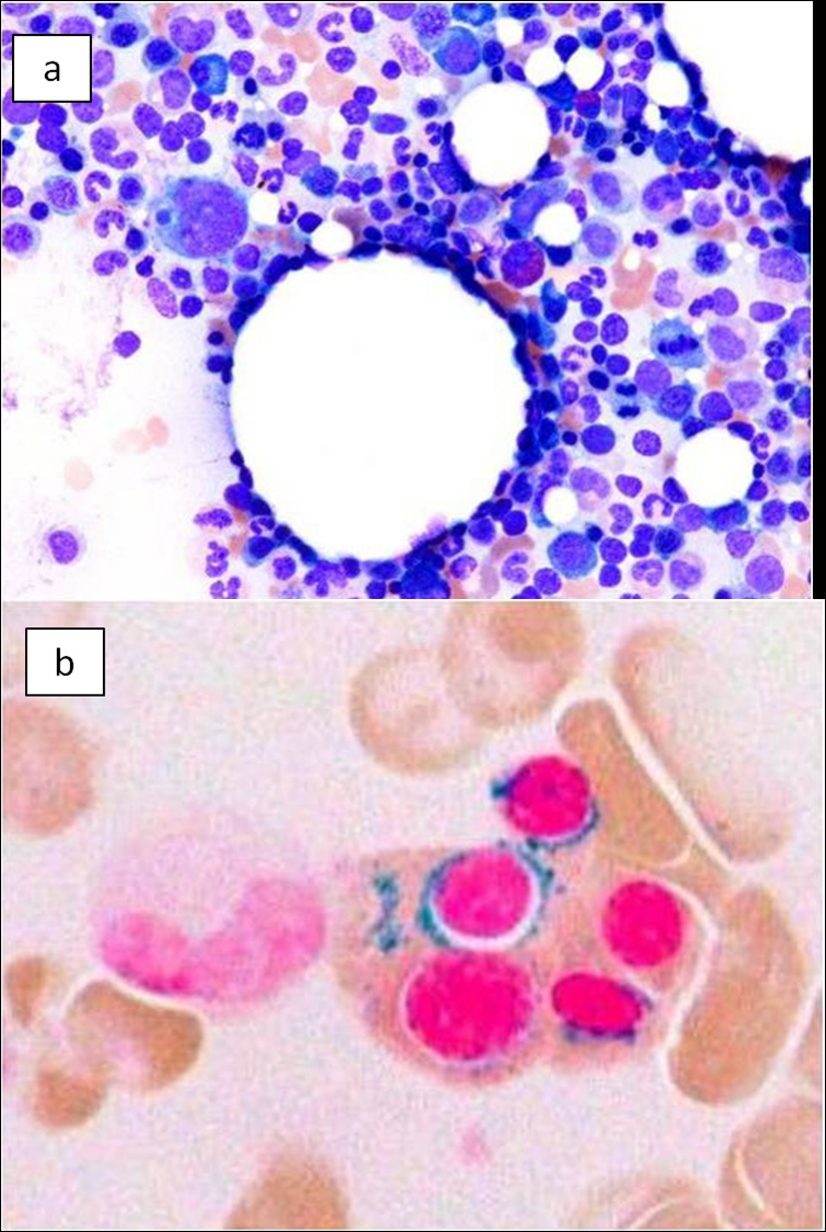
Bone marrow smear. (a) Bone marrow smear showed dysplastic change of three-lineage (erythroid, myeloid, and megakaryocyte) to be diagnosed as refractory cytopenia with *multilineage dysplasia and* ringed sideroblasts (RCMD-RS), WHO classification of MDS. (b) Ringed sideroblasts occupied 40% of erythroid precursors.

**Figure 4:**
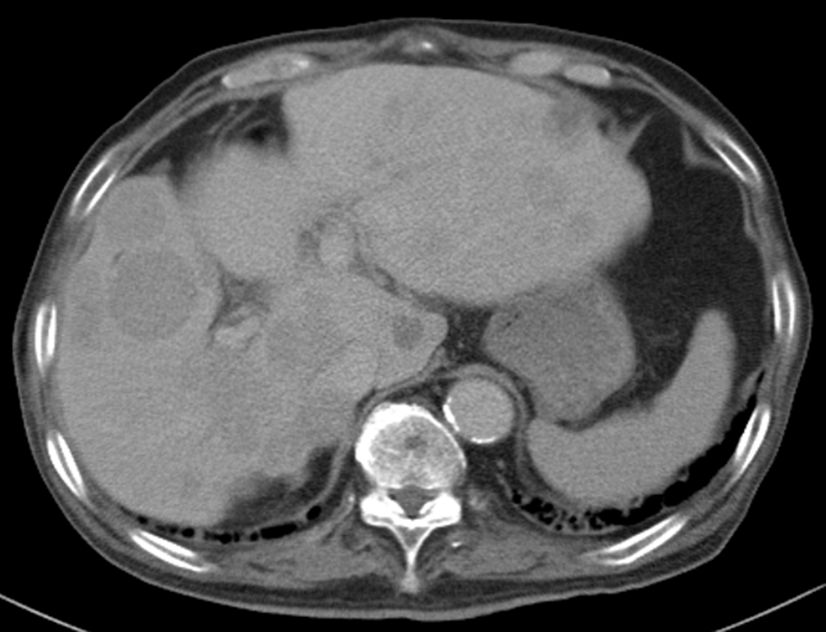
Computed tomography. Post-operative 2 years computed tomography at portal phase showing multicentric recurrence of hepatocellular carcinoma in the liver.

On the basis of these findings, the patient was diagnosed with hepatocellular carcinoma and liver iron overload. A resection of segment 8 of the liver was performed without complications. The pathological examination report showed a moderately differentiated HCC without vascular or lymphatic invasion with negative surgical margin. The Mallory stain revealed non-cirrhotic liver with a minimal fibrotic change around the portal vein area. The iron stain showed extensive intracellular iron deposition in the liver parenchyma (Fig. 2). A bone marrow aspiration was performed and revealed a dysplastic change in three lineages (erythroid, myeloid, and megakaryocytic), which were compatible with the condition of refractory cytopenia with multilineage dysplasia and ringed sideroblasts (RCMD-RS) (Fig. 3). We examined the germline mutations, namely, HFE-1, HJV-1, TFR2, SLC40A1, and FTH1, of the primary iron overload syndromes previously reported in Japanese patients [1], and all the germline mutations were found to have wild-type sequences. After the uncomplicated hospital stay and discharge, the patient was started on deferoxamine therapy, which successfully decreased the serum ferritin level to as low as 970 ng/mL. However, he was found to have a multicentric recurrence of HCC 2 years after the surgery on follow-up CT scan (Fig. 4), and underwent multiple transcatheter arterial chemoembolization (TACE). He is now 4 years after the surgery and on sorafenib tosilate 400 mg/day *per os*, which was started after the failure of 5th TACE with progressive disease.

## Discussion

Our patient developed secondary liver iron overload due to MDS. Iron overload occasionally occurs in MDS patients owing to ineffective erythropoiesis, followed by increased iron absorption and polytransfusion for anemia. As our patient did not have a history of transfusion or ferrotherapy, increased iron absorption may have played an important role in the occurrence of the iron overload.

Although an increased risk of HCC among patients with liver iron overload has been reported, only three cases of HCC associated with secondary iron overload have been reported (Table 1) [5-7] which include another case report of HCC arising in non-cirrhotic liver of MDS patient [7]. The excess risk of HCC in patients with cirrhotic HH has been estimated to be as high as 200-fold, but HCC in HH patients without cirrhosis remains to be rare [2]. This suggests that HH leads to HCC by causing cirrhosis of the liver. In other reports, a strong association was found between increased hepatic iron stores and HCC particularly among patients without cirrhosis [8], and iron was shown to induce DNA changes including p53 mutation in hepatocytes [9]. These are indicating a carcinogenic role of iron in the development of HCC, and regarding the fact that less than 2% of HCC develops in a non-cirrhotic liver and almost never in a normal liver [4], iron overload has contributed to the development and multicentric recurrence of HCC in the present case.

**Table 1:** Reported Cases of HCC Associated With Secondary Hemochromatosis

Author (Journal/year)	Primary disease	Age Gender	Cirrhosis	Blood transfusion	Treatment (outcome)
Barry et al (Lancet 1968)	Hereditary spherocytosis	64 Male	Present	None	Actinomycin D (Death at age of 65)
Tomas et al (Eur J Gastroenterol Hepatol 1997)	None Coalminer	61 Male	Absent Mild fibrosis	None (Iron containing water intake)	Right hepatectomy (No recurrence)
Chung et al (Hepatol Res 2003)	MDS (RA)	40 Male	Absent	180 concentrated erythrocyte transfusions	Deferoxamine TACE (No recurrence)
Current Report	MDS (RCMD-RS)	67 Male	Absent	None	Resection TACE (Progressive disease)

MDS: myelodysplastic syndrome; RA: refractory anemia; RCMD-RS: refractory cytopenia with multilineage dysplasia and ringed sideroblasts; TACE: transarterial chemoembolization.

### Conclusion

Although the prognosis of untreated hemochromatosis is poor, early diagnosis and iron removal therapy improve the survival rate and provide the normal life expectancy of patients [2]. Unfortunately, our patient had a multicentric recurrence of HCC despite the iron removal therapy. However, it demonstrated the importance of early detection and initiation of treatment of iron overload in preventing HCC in MDS patients, even among Asian population.

## Abbreviations

HCC: hepatocellular carcinoma
H&E staining: hematoxylin and eosin staining
TACE: transcatheter arterial chemoembolization
MDS: myelodysplastic syndrome
RCMD-RS: refractory cytopenia with ringed sideroblasts
CT: computed tomography
MRI: magnetic resonance imaging
HH: hereditary hemochromatosis
